# Potential synergistic effects of lenvatinib and aspirin in aortic dissection: a case report and literature review

**DOI:** 10.3389/fonc.2025.1520770

**Published:** 2025-04-22

**Authors:** Lan Luo, Hao Liang, Lin Yuan, Ya-Kun Wu

**Affiliations:** ^1^ Department of Pharmacy, Suining Central Hospital, Suining, Sichuan, China; ^2^ Department of Hepatobiliary Surgery, Suining Central Hospital, Suining, Sichuan, China

**Keywords:** lenvatinib, aspirin, adverse reactions, drug-drug interactions, hepatocellular carcinoma, aortic dissection

## Abstract

**Background:**

Although numerous anticancer drugs targeting vascular endothelial growth factor (VEGF) are commonly used in clinical practice, life-threatening drug-drug interactions (DDIs) involving these drugs are rarely reported.

**Case summary:**

A male patient had been taking aspirin for two years following a stroke. He was subsequently diagnosed with hepatocellular carcinoma (HCC) and initiated lenvatinib therapy. Shortly after, he developed lenvatinib-induced hypertension (191/102 mmHg) and was prescribed amlodipine for blood pressure control. At his first routine follow-up for HCC treatment, he was asymptomatic but was incidentally diagnosed with acute aortic dissection (AD). The patient declined endovascular treatment, and his AD lesions remained temporarily stable. Although lenvatinib is generally considered a safe and effective option for advanced HCC, this case raises concerns about a potential link between lenvatinib and aspirin in the development of AD due to their temporal association. This case highlights the need for increased clinical awareness regarding possible DDIs between these two drugs, particularly in patients with untreated acute AD.

**Conclusion:**

The concurrent use of lenvatinib and aspirin may increase the risk of AD in patients with cancer. To prevent life-threatening complications, patients receiving both therapies should be closely monitored and strictly adhere to treatment guidelines. For patients who decline invasive AD treatment, continued lenvatinib therapy might be a cost-effective option to improve prognosis, though the continuation of aspirin therapy requires careful consideration.

## 1 Introduction

With the rapid advancement of anticancer therapies, an increasing number of novel drugs have been introduced into clinical practice within a short period. Among them, vascular endothelial growth factor (VEGF) and platelet-derived growth factor (PDGF) receptors inhibitors, such as lenvatinib, have been widely adopted in cancer treatment. However, an increasing number of physicians have recorded unexpected adverse events (AEs) associated with these drugs (1). In particular, drug-drug interactions (DDIs) between newly developed anticancer drugs and pre-existing medications have raised concerns due to their potential to cause severe, life-threatening complications. Notably, evaluating these AEs through prospective randomized controlled studies remains challenging.

Aortic dissection (AD) is a rare but life-threatening disease. Recently, cases of AD linked to VEGF inhibitors have been reported by pharmacists, prompting increasing attention from clinicians (2). A 2024 retrospective study involving a large patient cohort reported a significantly elevated risk of AD in patients receiving VEGF inhibitor treatment (3). However, the complexity of clinical scenarios remains unanswered. Some patients undergoing VEGF treatment have a history of long-term medication use, particularly antiplatelet agents. Thus, the potential DDIs between VEGF inhibitors and antiplatelet therapy in AD may be complex.

Herein, we report an intriguing case of a patient incidentally diagnosed with acute abdominal AD while receiving lenvatinib therapy for advanced hepatocellular carcinoma (HCC) and long-term aspirin therapy. Additionally, we review existing literature on potential DDIs between lenvatinib and aspirin in the context of AD.

## 2 Case description

A 59-year-old man, asymptomatic at the time of presentation, was admitted to the Department of Vascular Surgery following the incidental detection of AD during a routine assessment for HCC treatment.

**Figure 1:**
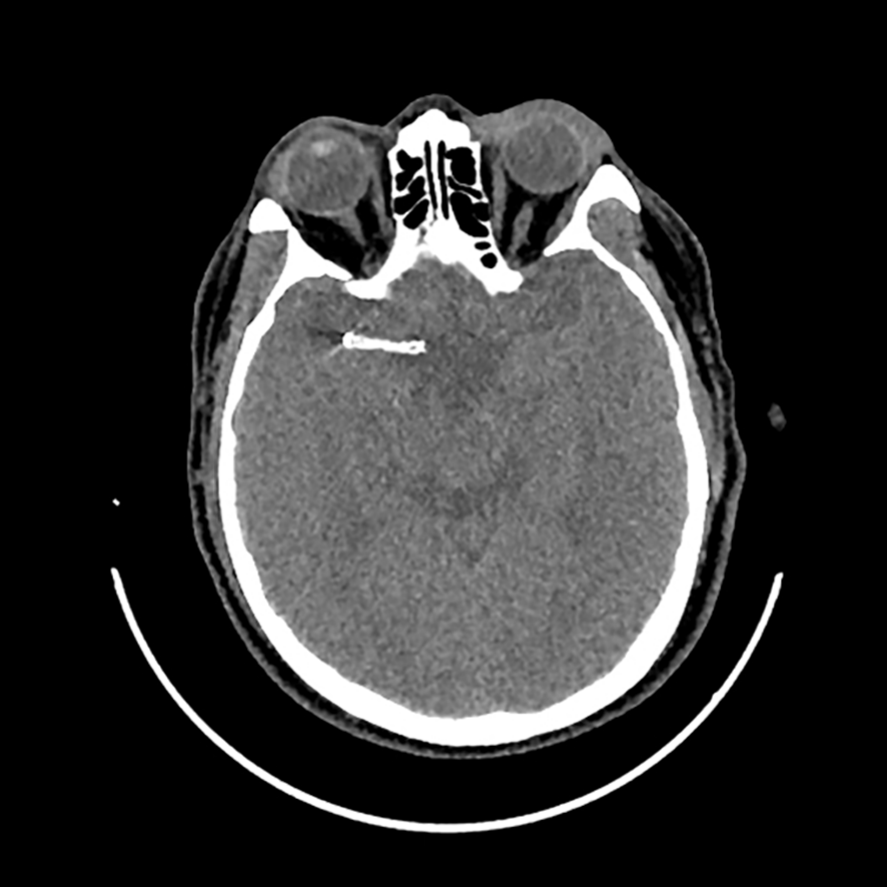
Implantation of stent in his middle cerebral artery in the computed tomography examination.

Two years prior, the patient had experienced a large-artery atherosclerosis-related stroke and had undergone stent implantation in his middle cerebral artery (**Figure 1**). Initially, he was prescribed dual antiplatelet therapy with ticagrelor and aspirin for six months. Due to post-angioplasty restenosis in the middle cerebral artery, dual antiplatelet therapy was extended to one year, after which he continued aspirin monotherapy (0.1 g once daily). The patient tolerated aspirin well without any adverse effects.

**Figure 2:**
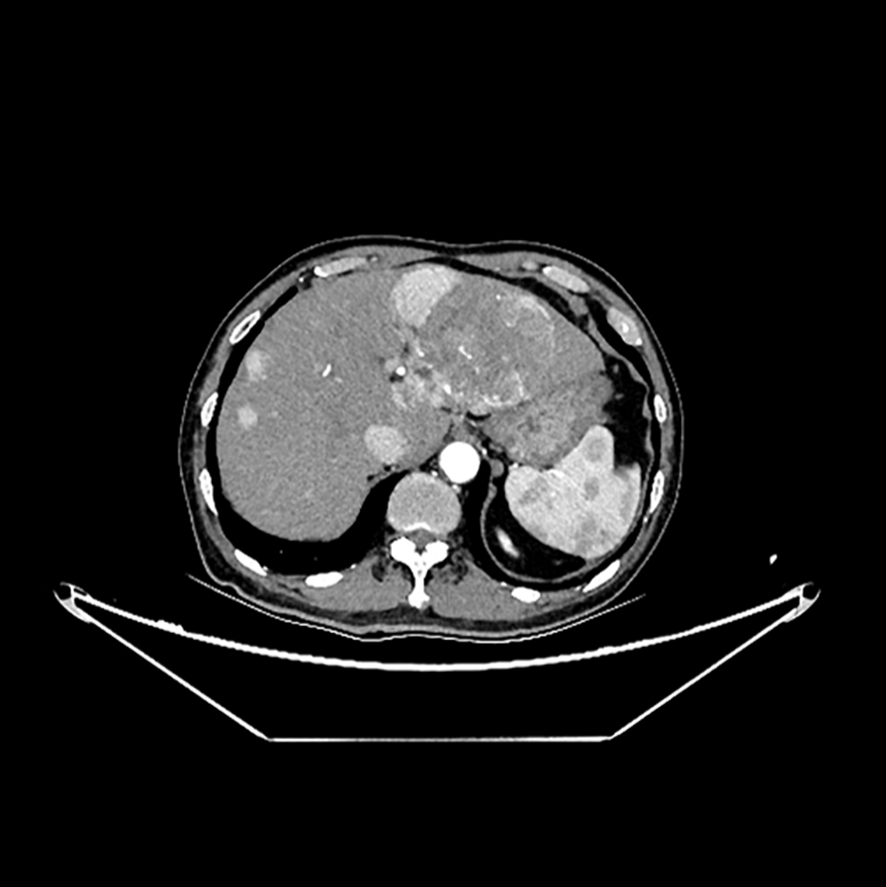
Tumor lesions in his left liver and normal outline of abdominal aorta in the computed tomography examination.

Approximately one month before admission, the patient was diagnosed with unresectable HCC and hepatitis C virus (HCV) infection. A computed tomography (CT) scan of the abdominal aorta at the time showed no abnormalities (**Figure 2**). He was subsequently discharged after starting treatment with aspirin and lenvatinib (8 mg once daily). Tramadol was prescribed for moderate-to-severe cancer-related pain. A few days after initiating lenvatinib and aspirin treatment, the patient developed hypertension (191/102 mmHg), which was potentially associated with lenvatinib use. He visited his physician and was prescribed amlodipine (5 mg once daily) to manage his blood pressure. His blood pressure stabilized within the normal range the following day. Until the day of admission, the patient did not report any new or worsening symptoms, including pain. Apart from lenvatinib-induced hypertension, no other side effects of lenvatinib were observed.

The patient had no significant medical history or common risk factors for AD. His blood sugar and lipid levels were within normal limits, and he had no history of arrhythmia, infective endocarditis, coronary heart disease, or heart failure. Additionally, there was no family history of rare diseases.

On admission, the patient was afebrile, with a body temperature of 36.5°C. His respiratory rate was 20 breaths/min, pulse rate was 64 beats/min, and blood pressure was 117/71 mmHg. He remained cooperative during the examination and reported no pain upon abdominal palpation. At the time of admission, he had been receiving aspirin for two years, lenvatinib for over one month, and amlodipine for nearly one month.

Several abnormal blood test results were observed. The patient’s hemoglobin level was 111 g/L (reference range: 130-175 g/L), while his platelet count remained within the normal range at 154 ×10^9^/L (reference range: 100-350). C-reactive protein level was elevated at 58.26 mg/L, and the D-dimer level was slightly increased at 0.57 µg/mL (reference range: 0–0.5). Activated partial thromboplastin time was within normal limits. The HCV antibody test was positive, whereas the HCV RNA test result was negative.

**Figure 3:**
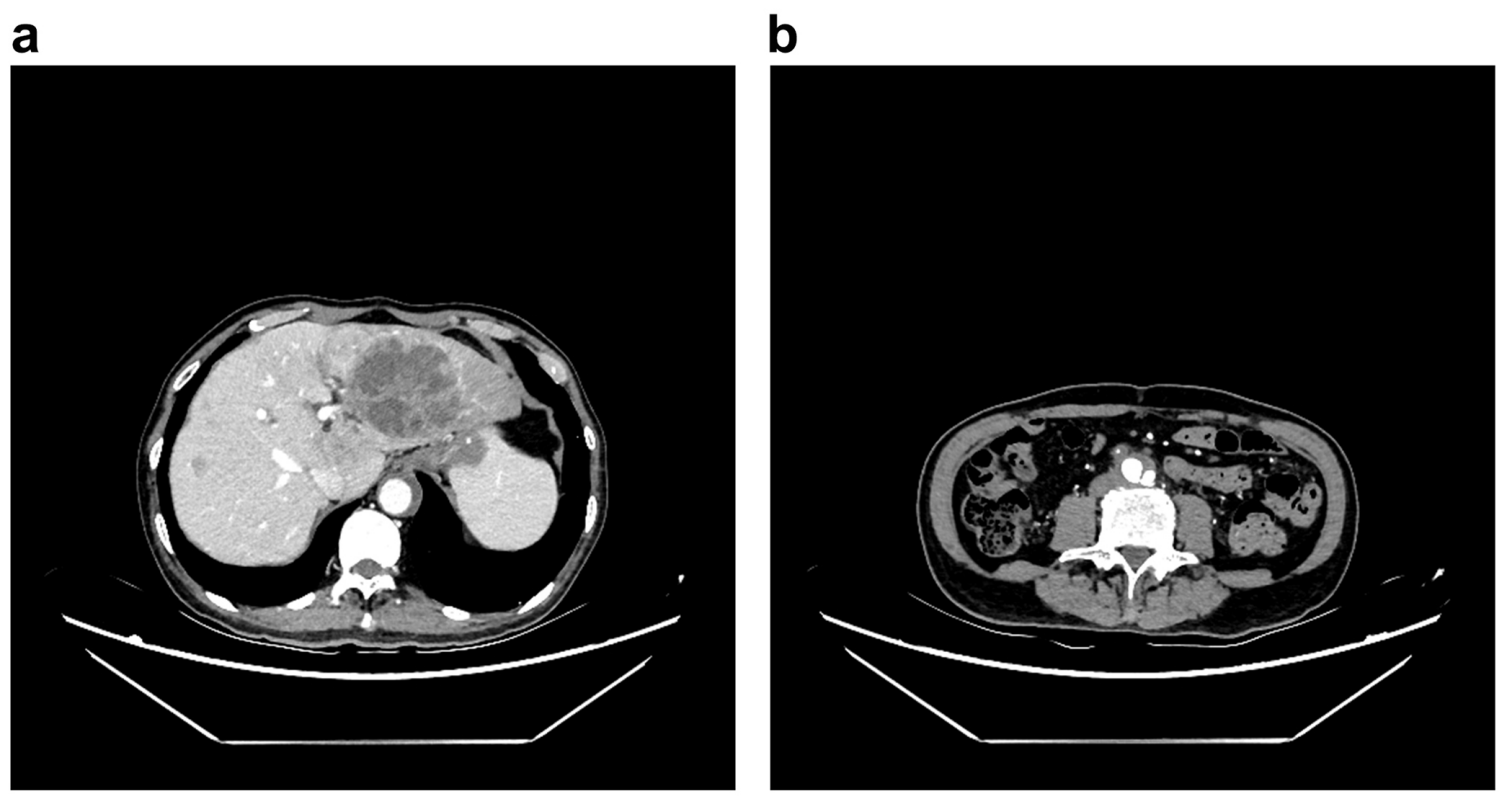
Signs of abdominal aortic dissection in the computed tomography examination, **(a)** abnormal outline of abdominal aorta in the level of liver, **(b)** outline of aortic dissection in the level of lower abdomen.

Electrocardiography showed no ischemic changes. CT imaging revealed a large tumor-like lesion in the left hepatic lobe, with multiple small lesions scattered throughout the liver (**Figure 2**). A Stanford type B AD was observed, extending from the infrarenal abdominal aorta to the common iliac artery (**Figures 3a, b**). Additionally, thrombi were detected in the inferior mesenteric and left common iliac arteries, and stenosis was observed in the celiac trunk.

The patient was diagnosed with acute abdominal aortic dissection (AAD). As he declined endovascular treatment, optimal medical therapy was initiated and appeared to be effective. Lenvatinib, amlodipine, and aspirin were temporarily discontinued during hospitalization, and his blood pressure remained stable throughout his admission. Follow-up CT imaging one week later revealed that the AAD lesions remained unchanged. The patient was subsequently discharged. However, post-discharge, the concurrent use of lenvatinib and aspirin, along with their potential DDIs, remained a concern for his physicians, who strongly advised the patient to keep regular follow-up evaluations. A review of similar cases and relevant literature has been conducted (**Table 1**).

**Table 1:** Patients without history of hypertension diagnosed with AD after received VEDFI treatments for cancer.

Article	Age (yeas)	Sex	Type of cancer	Stage of cancer	Type of VEGFI	Type of AD	BP at admission (mmHg)	Treatment for AD	Drugs in the following treatments	OS from diagnosis of AD (month)
(Jiang, Li et al. 2020) (13)	58	Male	Lung squamous cell carcinoma	IV	Anlotinib	Stanford type A	180/120	Stent‐graft intervention	Anticoagulant, Nitroglycerin, Nivolumab	2
(Takada, Yasui et al. 2018) (10)	66	Male	Metastatic renal cell carcinoma	IV	Sorafenib and Axitinib	Stanford type A	130/ N/A	Non-invasive treatment	Carvedilol, Nivolumab	N/A
(Tsuchiya, Ogawa et al. 2018) (11)	65	Male	Advanced hepatocellular carcinoma	BCLC C	Sorafenib	Stanford type A	128/54	Ascending aorta replacement	ACEI, 5- fluorouracil, Cisplatin	34
(Hatem, Bebawi et al. 2017) (14)	63	Female	Recurrence of gastrointestinal tumor	N/A	Sunitinib	Stanford type A	N/A	Aortic replacement	Sunitinib	recovered
(Formiga and Fanelli 2015) (9)	68	Male	Metastatic renal clear cell carcinoma	IV	Sunitinib	Stanford type B	210/120	Endoprosthesis	Metoprolol, Temsirolimus	Over 2
(Serrano, Suárez et al. 2010) (12)	77	Female	Renal cell carcinoma	IV	Sorafenib	Stanford type B	200/106	Non-invasive treatment	β-blocker, ACEI, Diuretic	N/A

**AD**: Aortic dissection; **VEGFI**: Vascular endothelial growth factor tyrosine kinase inhibitor; **BCLC**: Barcelona Clinic Liver Cancer staging; **BP**: Blood pressure; **OS**: Overall survival; **ACEI**: Angiotensin converting enzyme inhibitors; **N/A**: Not available.

## 3 Discussion

We report a case of a patient who was incidentally diagnosed with AAD after receiving lenvatinib and aspirin therapy. AAD is a life-threatening vascular disease. However, cases of AAD induced by the combined use of lenvatinib and aspirin in patients without a history of hypertension are rare.

In this case, the patient developed AAD approximately one month after initiating lenvatinib and aspirin therapy despite well-controlled lenvatinib-induced hypertension. The temporal association between these medications and AAD suggests a potential interaction. We hypothesize that aspirin use may have contributed to the onset and progression of AAD. While aspirin plays a crucial role in reducing the risk of stroke recurrence (4), its use in untreated AAD may inadvertently accelerate disease progression and increase the risk of rupture. This presents a complex clinical challenge, emphasizing the need for a careful risk-benefit assessment when considering aspirin therapy in such patients.

Several VEGF inhibitors are used in clinical practice, and their association with aortic dissection has been documented. One study reported that 49 out of 16,441 patients (0.3%) treated with VEGF inhibitors developed AD, a rate 30 times higher than in those who did not receive VEGF inhibitor therapy (5). According to the World Health Organization’s centralized database of drug reactions (2), bevacizumab was associated with the highest risk of promoting AD (222 cases, 44.9%), while lenvatinib-related AD was reported in 11 cases (2.2%).

Lenvatinib has demonstrated safety and efficacy in the treatment of advanced HCC (6). Its mechanism of action involves inhibiting angiogenesis and tumor proliferation while also exerting immunomodulatory effects (7). A preclinical study in mice suggested that the combination of lenvatinib and aspirin may have a synergistic anticancer effect against HCC (8). However, clinical evidence supporting this synergy in humans remains limited. Conversely, some VEGF inhibitors (9–14), such as anlotinib have been associated with AD when co-administered with anticoagulants. Given that lenvatinib inhibits PDGF receptors, potential DDIs with aspirin warrants further investigation.

One hypothesis suggests that lenvatinib-induced hypertension may contribute to vascular endothelial injury (15), while also impairing the formation of nutrient vessels within the arterial wall. This could lead to microscopic tears in the aortic intima (16). Simultaneously, aspirin inhibits platelet aggregation and adhesion, potentially impairing vascular repair mechanisms. The combined use of aspirin and lenvatinib may, therefore, exacerbate endothelial damage, increasing the risk of AD. Although this hypothesis is based on limited data, clinicians should exercise caution when prescribing lenvatinib alongside aspirin. Close monitoring for AD in these patients is recommended to mitigate potential risks.

The pathological mechanisms underlying AD remain unclear. Factors such as pre-existing vascular disease, genetic predisposition, and blood pressure variability may contribute to its development (17, 18). In this patient, there was no documented history of vascular disease or genetic predisposition. However, fluctuations in blood pressure may have played a role in the onset of AD. Lenvatinib-induced hypertension was effectively controlled upon discontinuation of lenvatinib. During follow-up, when lenvatinib was reintroduced for the treatment of HCC, amlodipine was prescribed to manage hypertension associated with its use.

Lenvatinib-induced hypertension requires careful management, as it is the most common adverse effect, occurring in 42.2% of patients (6). Calcium channel blockers and potassium-sparing diuretics can help control this hypertension (19). However, the risk of AD in these patients remains unpredictable (10, 11). Current guidelines recommend β-Receptor blockers as first-line agents for managing lenvatinib-induced hypertension (9, 20). In cases of uncontrolled hypertension, adjusting lenvatinib dosage or discontinuing treatment should be considered (21). However, as lenvatinib is an effective treatment for advanced HCC (6), its withdrawal could reduce survival time, presenting a complex clinical challenge that warrants further investigation.

The use of aspirin in this patient requires careful cardiovascular risk assessment. Currently, no high-quality evidence supports either the continuation or discontinuation of aspirin in patients with AD. Existing data on the effects of antithrombotic therapy in patients with AD remain inconclusive (22, 23). In this patient, aspirin treatment was discontinued during the acute phase of AD. However, once the AD lesions stabilized and considering the patient’s reliable adherence to follow-up, aspirin therapy was reconsidered—despite the patient’s refusal to undergo invasive treatment for AD. In a case report, antiplatelet therapy might not significantly increase cardiovascular risks (24). Using newer antiplatelet agents with a lower cardiovascular risk might be more beneficial.

Regarding prognosis, the occurrence of AAD can negatively impact clinical outcomes (25). Many patients diagnosed with advanced cancer have limited treatment options, and discontinuation of VEGF inhibitors can lead to disease progression (13). However, in clinical practice, physicians often recommend stopping VEGF inhibitors following an AD diagnosis (9–14), even in patients who have undergone invasive treatment. Consequently, the tumor’s malignancy grade may considerably influence the patient’s overall prognosis.

## 4 Conclusion

Patients with cancer receiving lenvatinib and aspirin should be closely monitored due to the potential risk of AD. The combination of these therapies may contribute to AD development, particularly in those with lenvatinib-induced hypertension, which could serve as a predisposing factor. The concurrent use of lenvatinib and aspirin may lead to microscopic tears in the aorta intima, increasing the risk of AD rupture. For patients who decline invasive treatment for AD, continuing lenvatinib may be a cost-effective strategy for improving prognosis. However, the safety of aspirin therapy in such cases requires further evaluation. Clinicians should remain vigilant regarding potential DDIs between lenvatinib and aspirin, as a deeper understanding of these interactions is crucial for optimizing patient outcomes.

## Data Availability

The raw data supporting the conclusions of this article will be made available by the authors, without undue reservation.
